# A Review of the Cognitive Effects Observed in Humans Following Acute Supplementation with Flavonoids, and Their Associated Mechanisms of Action

**DOI:** 10.3390/nu7125538

**Published:** 2015-12-09

**Authors:** Lynne Bell, Daniel J. Lamport, Laurie T. Butler, Claire M. Williams

**Affiliations:** School of Psychology and Clinical Language Sciences, University of Reading, Earley Gate, Whiteknights, Reading RG6 6AL, UK; l.bell@pgr.reading.ac.uk (L.B.); d.j.lamport@reading.ac.uk (D.J.L.); l.t.butler@ reading.ac.uk (L.T.B.)

**Keywords:** flavonoid, polyphenol, cognition, effect size, mechanism

## Abstract

Flavonoids are polyphenolic compounds found in varying concentrations in many plant-based foods. Recent studies suggest that flavonoids can be beneficial to both cognitive and physiological health. Long term flavonoid supplementation over a period of weeks or months has been extensively investigated and reviewed, particularly with respect to cognitive ageing and neurodegenerative disease. Significantly less focus has been directed towards the short term effects of single doses of flavonoids on cognition. Here, we review 21 such studies with particular emphasis on the subclass and dose of flavonoids administered, the cognitive domains affected by flavonoid supplementation, and the effect size of the response. The emerging evidence suggests that flavonoids may be beneficial to attention, working memory, and psychomotor processing speed in a general population. Episodic memory effects are less well defined and may be restricted to child or older adult populations. The evidence also points towards a dose-dependent effect of flavonoids, but the physiological mechanisms of action remain unclear. Overall, there is encouraging evidence that flavonoid supplementation can benefit cognitive outcomes within an acute time frame of 0–6 h. But larger studies, combining cognitive and physiological measures, are needed to strengthen the evidence base.

## 1. Introduction

Flavonoids are a class of organic polyphenolic compounds found in varying concentrations in plant-based whole foods such as berries, tea, cocoa, soybeans, and grains. Herbal extracts are also commonly prepared from the leaves, bark, or berries of these plants to provide a more concentrated flavonoid source. There are several subclasses of flavonoid, including flavanols, flavonols, anthocyanidins, flavones, flavanones and isoflavones. Flavonoids often naturally occur in polymer or conjugate form depending on the food type. A detailed overview of flavonoid structure is provided by Beecher [1], however a brief description of the main flavonoid forms relevant to this review is contained in Table 1.

**Table 1:** Flavonoid subclasses and their naturally occurring forms.

Flavonoid Subclass	Food Source	Additional Naturally Occurring Forms
Anthocyanidins; e.g., cyanidin, delphinidin	Berries	Anthocyanidins may occur in methylated form, e.g., malvidin. All anthocyanidins conjugate with saccharide (sugar) groups to form anthocyanins *, e.g., chrysanthemin
Flavanols; e.g., catechin	Tea, cocoa	All flavanols are isomers, polymers or gallated conjugates of catechin, e.g., epicatechin *, epigallocatechin gallate (EGCG) *
Flavonols; e.g., kaempferol *, quercetin *	Fruits, vegetables	Flavonols may occur in methylated form, e.g., isorhamnetin * and/or conjugate with saccharides
Flavones; e.g., apigenin, luteolin	Cereals, herbs	Flavones conjugate with saccharides
Flavanones; e.g., naringenin	Citrus fruits	Flavanones may occur in methylated form e.g., hesperetin *, and/or conjugate with saccharides, e.g., hesperidin *, narirutin *
Isoflavones; e.g., daidzein, genistein	Soya beans, peanuts	Isoflavones may occur in methylated form and/or conjugate with saccharides

* Flavonoid compounds included in this review.

Flavonoids have been well documented to elicit health benefits by reducing the risk factors associated with cardiovascular disease, diabetes and stroke [2,3]. Over recent years interest has also grown in their ability to elicit cognitive benefits. As such, long term chronic supplementation with flavonoids has been investigated extensively, particularly with respect to cognitive ageing and related neurodegenerative disorders [4,5,6,7,8,9,10,11,12,13,14,15,16,17,18,19,20]. Less attention has been given to the acute effect of flavonoids on cognitive outcomes, *i.e.*, within the immediate 0–6 h post ingestion. This review focuses on the limited, but increasing body of evidence for the cognitive benefits of acute flavonoid supplementation. Immediate cognitive enhancement is often desirable in academic and work environments, such as during an exam or assessment. Flavonoids may be useful alternatives to stimulants such as caffeine in such situations. In addition to these potential practical benefits, acute studies are important in understanding the full range of effects that flavonoids may elicit, and their mechanisms of action. A number of recent acute supplementation studies in humans are reviewed here.

## 2. Method

A search of Google Scholar, Pubmed, Web of Science and PsychInfo was performed using the keywords flavonoid, polyphenol, memory, and cognition (including truncated forms). The studies selected for inclusion have all been subject to peer and/or editorial review, or form part of a published doctoral thesis. For this reason, conference abstracts have been omitted. Also included is a recent study from the University of Reading, Nutritional Psychology Research Group, which is currently under review [21].

Flavonoids are typically administered in food form rather than as pure compounds, therefore the studies reviewed here have been categorised according to the food source. As shown in Table 1 and Table 2, the flavonoid subclass or subclasses present often differ according to the food source. This suggests the potential for differences in cognitive effect between food groups, and further supports this method of categorisation. The subclass and dose of flavonoids administered, the cognitive domains affected, and the effect size of the response are discussed, along with associated physiological mechanisms of action. Where possible, Cohen’s d effect sizes have been calculated using a published meta-analysis method [22]. Cohen’s d is a standardised measure that describes effect size in terms of the number of standard deviations between group means. Values of d equal to 0.2, 0.5 and 0.8 correspond to small, medium and large effect sizes, respectively. In the majority of studies reviewed, post-intervention scores have been compared with those obtained for a placebo or control (d), or if this information was not available then post-intervention scores were compared with baseline values (d_b_).

**Table 2:** Average flavonoid compositions for different food types.

Food Type	Flavonoid Composition (mg/100 g)				
	Anthocyanidins	Flavanols	Flavanones	Flavones	Flavonols
Apples (whole)	1.59	9.29	0.00	0.12	4.15
Blackcurrants (whole)	157.78	1.17	-	0.00	11.46
Blueberries (whole, cultivated)	163.30	6.69	0.00	0.20	10.63
Cherries (whole, red)	33.44	4.13	-	0.00	2.43
Cocoa (powdered)	-	52.73	-	-	2.03
Ginkgo biloba (EGb 761) *	-	-	-	-	-
Grapes (whole, Concord)	120.1	2.14	-	-	3.11
Green tea (brewed)	-	132.81	-	0.30	4.82
Oranges (whole)	-	0.00	42.57	0.19	0.73

Data obtained from the USDA database for the flavonoid content of selected foods [23]; * Ginkgo biloba EGb 761 is a standardised extract that contains 24% flavonols in saccharide conjugate form [24].

## 3. Cognitive Effects Following Acute Flavonoid-Rich Food Supplementation

### 3.1. Fruit Supplementation

#### 3.1.1. Berry Anthocyanins (Blackcurrant, Blueberry, Cherry, Cranberry, Grape)

As shown in Table 2, berries contain a range of different flavonoid subclasses, but they are typically richest in anthocyanins. Initial berry studies predominantly investigated the cognitive effects of whole fruit. For example, Dodd [25] demonstrated improved accuracy on a letter memory task (measuring working memory) following freeze dried whole blueberries (200 g fresh equivalent, 631 mg anthocyanidins), in 19 young adults at a postprandial time point of 5 h (*d* = 0.57). The study employed a double blind, crossover design with an energy matched control condition. No effects were observed at an earlier time of 2 h or for other measures of executive function, memory, or mood. For a subset of participants, blood samples taken 1 h postprandially revealed a trend towards increased plasma levels of brain-derived neurotrophic factor (BDNF) in the blueberry condition. Unfortunately, cognition was not measured at this time point so it is impossible to say whether the neurochemical changes are related to the cognitive outcome. In the same study, older adults’ BDNF values decreased from baseline for both blueberry and placebo conditions, but the decrease at the 1 h time point was attenuated in the blueberry condition. These older adults (*n* = 18) showed improved performance on an immediate word recognition task at both 2 h (*d* = 0.44) and 5 h (*d* = 0.69) postprandially, but no improvements in executive function or mood were observed.

A later study by Whyte and Williams [26], using fresh whole blueberries (200 g, 143 mg anthocyanins), investigated cognitive effects in children. They found no effects at 2 h for a range of executive function tasks, but did observe a significant improvement in delayed word recall using the Rey auditory verbal learning task (RAVLT) (*d* = 0.74). This was a small, crossover study with only 14 participants. As no baseline measures were taken, variations in performance across test days may have reduced the statistical power. Nevertheless, the medium effect size for the RAVLT provides good evidence for positive effects of blueberry flavonoids in children. Whyte, Schafer and Williams [27] conducted a larger (*n* = 21) double-blind, placebo-controlled, crossover study investigating the cognitive effects of 2 separate blueberry doses (127 mg and 253 mg anthocyanins), again in children. The highest dose resulted in significant improvements in immediate word recall after 1.25 h (*d* = 0.80), and in delayed word recognition after 6 h (*d* = 0.78). Improved accuracy was observed during a flanker interference task after 3 h, although only for cognitively demanding incongruent trials (*d* = 0.78). However, reaction times for a Go-NoGo measure of inhibition revealed significantly faster performance following the placebo compared with the blueberry interventions.

The positive blueberry effects in older adults and children appear to be focussed on episodic memory, whereas improvements in executive function are more consistent in young adults. The differences in cognitive domains may be an artefact of the small sample sizes, but could also be indicative of age differences in the capacity for improvement in underlying neuronal structures. For example, hippocampal function may be more receptive during development in childhood and decline in old age, whilst frontal regions associated with executive function may be more sensitive in young adulthood. It is noteworthy that neurochemical changes in BDNF were apparent after 1 h, yet distinct time points for memory effects emerged at 1.25–2 h and 5 h, but not at an intervening 3 h time point. At this stage only BDNF trends have been observed, and not directly in association with cognitive changes. Although it is perhaps premature to comment on the relationship between acute changes in cognition and BDNF, it has nevertheless been posited that flavonoid induced increases in BDNF may facilitate stronger memory encoding [27]. Possible mechanisms of action are discussed below. Overall, the timings of cognitive effects are likely to be related to the digestion, absorption and metabolism of flavonoids, but further mapping of cognitive and physiological observations is required in order to resolve inconsistencies within the current observations.

The flavonoid content of blueberries is known to vary widely depending on growing, processing and storage conditions [28,29]. The same 200 g quantity of whole blueberries used in the first two studies [25,26] described above showed extreme differences in flavonoid content (631 mg anthocyanidins and 143 mg anthocyanins respectively). This highlights the importance of analysing fresh fruits for their flavonoid content when conducting an intervention. It is also important to note that compositional analysis of anthocyanins typically (but not exclusively) involves the removal of saccharide conjugates prior to quantification; therefore anthocyanin content is often reported as anthocyanidin equivalent. This difference is critical when comparing doses between studies. For example, a berry intervention reported to contain 100 mg cyanidin may actually contain 156 mg chrysanthemin (a saccharide of cyanidin). Some studies reviewed here appear to use the terms anthocyanins and anthocyanidins interchangeably without acknowledging this distinction, making it unclear whether a reported anthocyanin dose is actually referring to an equivalent anthocyanidin dose.

Similarly to blueberries, blackcurrants are a rich source of anthocyanins. Watson *et al*. [30] conducted a double-blind, controlled crossover trial of two blackcurrant extracts (cold-pressed juice or freeze-dried powder). Improved attention compared with an energy-matched control was observed in 36 young adults during a 70-min-long, cognitively fatiguing battery, beginning 1 h postprandially. Specifically, declining accuracy on a rapid visual information processing (RVIP) task was attenuated after taking the powdered extract (*d* = 0.10, *d* = 0.47, *d* = 0.47, *d* = 0.49, *d* = 0.49, *d* = 0.59, *d* = 0.56, measured for 7 task repetitions; once every 10 min). Similarly, a slowing of reaction time on a digit vigilance task following both blackcurrant and placebo interventions was attenuated after taking the juiced extract (*d* = 0.60, *d* = 0.73 and *d* = 0.60 for the 1st, 4th and 7th repetitions of the task, respectively). No effects were observed for the Stroop test (a measure of inhibition and attention), or for subjective measures of mood and mental fatigue. The total polyphenol content of the two extracts were matched at 525 mg/60 kg bodyweight. However, the anthocyanin content differed slightly; 483 mg/60 kg bodyweight for the powder and 467 mg/60 kg for the juice. This difference was also reflected in the analysis of plasma anthocyanin levels, which were observed to be higher following consumption of the powdered extract compared with the juice. Interestingly, the juice but not the powder was observed to inhibit platelet monoamine oxidase (MAO) and to attenuate blood glucose decline over the duration of the 70-min task battery. This study suggests that the way an extract is prepared may influence cognitive and physiological outcomes, however as different blackcurrant cultivars were used for each extract, the contrasting observations may simply represent compositional differences such as the ratio of flavonoid subclasses present. For the juiced blackcurrant extract, MAO inhibition and blood glucose regulation emerge as possible mechanisms of action further to the neurochemical changes observed for blueberries. The significant cognitive effects were observed for tests of executive function (RVIP and to some extent vigilance) which is consistent with the executive function benefits reported in healthy young adults following blueberry anthocyanins.

In a double-blind crossover intervention study, Hendrickson and Mattes [31] investigated whether an acute dose of grape juice would mitigate deficits in mood and cognition that commonly occur following a large meal. Approximately 600 mL (10 mL/kg), containing around 580 mg anthocyanins, was administered to young adult smokers along with a standardised lunch. Smokers were selected on the rationale that this population have an increased propensity to oxidative stress, and because smoking abstinence can exaggerate the post-meal dip in cognitive or affective state, thus this population may be more sensitive to the effects of flavonoids than healthy non-smokers. This was a large study (*n* = 35) with considerable statistical power, yet no significant effects of grape juice were observed 1 h postprandially when compared to an energy matched placebo condition. Mood ratings for positive mood states (pleasure, arousal and vigor) were observed to decline under both grape and placebo conditions, similarly ratings of negative mood states (confusion and fatigue) increased under both conditions. Although mood generally declined, word fragment completion task performance did not significantly change over time in either condition. It is unfortunate that performance on only one cognitive domain was examined (implicit memory), which is an area that has not previously been considered with respect to flavonoid intervention. Studies may be more likely to observe effects on traditional measures of explicit memory and executive function.

Recently, Caldwell *et al*. [32] published their investigations into the effects of cherry flavonoids. Following administration of 300 mL cherry juice (55 mg anthocyanins) to younger adults (*n* = 6), older adults (*n* = 5) and older adults with mild cognitive impairment (*n* = 5), tests of executive function, speed of processing, and verbal learning and memory were performed at baseline and 6 h postprandially. At 6 h, the older adults displayed improved task switching performance compared to baseline (*d_b_* = 0.75). No other cognitive effects were observed. The authors attribute this single effect to type 1 error, citing attrition of participants in that group as a likely cause. However the small sample size in all groups suggests that the whole study is likely to be severely underpowered. The lack of an energy matched, low flavonoid control condition is also cause for concern; a second crossover condition only administered the same juice in three separate 100 mL aliquots each consumed 1 h apart. No cognitive effects were observed relative to baseline following consumption of the juice in these consecutive smaller doses. A further problem may be the intervention itself; the anthocyanin content of the cherry juice appears very low compared to some of the above studies. A considerably larger, controlled study is therefore needed to determine if cherry anthocyanins elicit acute cognitive effects similar to those of other anthocyanin-rich fruits.

#### 3.1.2. Citrus Hesperidin (Orange)

As with berries, citrus fruits contain several different flavonoids, but they are richest in the flavanone hesperidin. In a crossover intervention study, Lamport *et al.* [21] supplemented 24 young adults with a commercially available flavanone-rich orange juice (70.5 mg total flavonoids; 42.15 mg hesperidin, 17.25 mg naringin, 6.75 mg narirutin) or an energy matched control. An extensive battery of tasks including measures of vision, episodic memory, processing speed, working memory, and other executive functions were performed 2 h postprandially. Significant improvements when compared to both control and baseline were observed only for the digit symbol substitution task (DSST), a measure of psychomotor processing speed (*d* = 0.30). An additional group of participants underwent fMRI assessment using arterial spin labelling (ASL). Increased cerebral blood flow (CBF) was observed at 2 h but not 5 h postprandially. However, as cognition was not measured directly in conjunction with CBF (and not at all at 5 h) it remains unclear whether the observed improvements in processing speed are causally related to the CBF changes. The flavonoid dose used in this study was low, particularly when compared with the typical doses of other flavonoid subclasses reviewed here. If flavonoid effects are dose dependent then a higher dose may have elicited increased cognitive benefit. Indeed, a subsequent study by Alharbi *et al.* [33] used orange juice fortified with additional flavanone rich orange pulp, to achieve a greater total flavonoid content (272 mg; 220.46 mg hesperidin, 34.54 mg narirutin). Middle aged adults (*n* = 24, 30–65 years) showed improved psychomotor performance on a finger tapping task at both 2 h (*d* = 0.87) and 6 h (*d* = 0.62). Improvements in attention and general executive function, as measured by a continuous performance task (CPT), were observed at 6 h (*d* = 0.58). The orange juice was observed to attenuate a decline in subjective alertness throughout the testing period compared to a sugar-matched control.

From this limited research, orange juice appears to benefit psychomotor performance across age groups at a time point also associated with increased CBF. Higher doses appear to elicit greater effect sizes for psychomotor performance and provide additional benefits in executive function, suggesting that flavanone effects are dose-dependent.

#### 3.1.3. Quercetin and Epicatechin (Apple)

Bondonno *et al.* [34] conducted a crossover intervention study investigating the cognitive effects of whole fresh apple (184 mg quercetin and 180 mg epicatechin), spinach or a combination of the two. It was hypothesised that the nitrate content of spinach and the flavonoids in apple would both augment plasma nitric oxide (NO), although through distinct mechanisms. Increased NO status has been previously associated with vasodilatory related increases in blood flow, which the authors predicted would lead to a beneficial cognitive outcome following all three intervention conditions. Cognition and mood were measured 2.5 h postprandially, using the Cognitive Drug Research (CDR) assessment battery and Bond-Lader mood scales. Even though significant increases in plasma NO were observed for the apple, spinach and combined conditions when compared with a control condition, all cognitive outcomes remained non-significant. This included factors for working memory and attention derived from the CDR results. This was a reasonably well powered study with 30 middle aged participants (mean 47 years), so the lack of cognitive findings suggests that augmented NO status may not affect cognition. If other mechanisms are responsible for previously observed cognitive effects of flavonoids, then it may be that apple flavonoids are not particularly effective, or that the peak time for observing apple flavonoid effects was missed by the study. The rationale for testing 2.5 h postprandially is not given in the paper, but may relate to previous NO observations rather than the absorption and metabolism of apple flavonoids.

### 3.2. Cocoa Supplementation (Epicatechin)

Cocoa is a rich source of the flavanol epicatechin, however it also contains caffeine and theobromine that have known psychoactive properties. Not all of the cocoa studies reviewed here have matched for these potential confounds across experimental conditions. Scholey *et al.* [35] carried out a double-blind crossover study of 30 young adults (age 18–35), comparing two acute doses of chocolate milk with a fully matched control. Beginning 90 min postprandially, participants performed 6 consecutive repetitions of a 10 min task battery, designed to be cognitively fatiguing. A 520 mg dose of cocoa flavanol resulted in significant improvements in working memory on a serial 3s subtraction task at all 6 time points (*d* = 0.57, *d* = 0.71, *d* = 0.50, *d* = 0.64, *d* = 0.50 and *d* = 0.41 respectively) relative to the control. A 994 mg dose resulted in significant improvements for the first 4 repetitions on the same serial 3s task (*d* = 0.44, *d* = 0.52, *d* = 0.41 and *d* = 0.67 respectively). However, no improvements were observed whilst performing the more difficult serial 7s task. Only the high dose was reported to improve reaction time on an RVIP attention task, with significant improvements observed during the 3rd and 4th repetitions (*d* = 0.35 for each repetition). Conversely, only the low dose improved self-reported levels of mental fatigue, with significant attenuation of fatigue across all but the 3rd repetition (*d* = 0.39, *d* = 0.37, *d* = 0.30, *d* = 0.27 and *d* = 0.30 respectively). Overall the low dose was observed to be effective over more time points than the high dose for serial 3s and conveyed greater benefits in terms of counteracting mental fatigue. The high dose resulted in some additional reaction time benefits but also incurred cognitive costs with an increased error rate observed during the serial 7s task. The authors conclude that lower doses of cocoa flavanol may be more effective but do not proffer an explanation as to why this might be the case.

In contrast, a similar study of older adults by Pase *et al.* [36] failed to observe any acute effects of a water based chocolate drink on cognition or mood. The doses were 250 mg and 500 mg cocoa flavanol, and a fully matched control. The study used a broad spectrum of tasks from the CDR assessment battery and a range of Bond-Lader mood visual analogue scales. Testing was performed at 1 h, 2.5 h, and 4 h time points. The inconsistencies between the two studies in population, testing time point, and dose make it difficult to draw conclusions from the opposing outcomes. However, a notable methodological difference was that Pase *et al.* [36] provided a light lunch 1.5 h after the intervention. The effects of flavonoids may have been masked by stronger macronutrient effects, such as the neuronal energy provided by carbohydrate ingestion.

In support of Scholey *et al.* [35], Field *et al.* [37] observed improved performance in visual spatial working memory (VSWM) (*d* = 0.35) and choice reaction time (CRT) (*d* = 0.16) in 30 young adults (age 18–30), following a 773 mg high dose of cocoa flavanols relative to a low flavanol white chocolate control. Testing was performed 2 h postprandially. Visual function also benefited; significant improvements in visual contrast sensitivity and time to detect motion direction were observed following the high dose. However, caffeine and theobromine were not matched across the two conditions and baseline measurements were not recorded before administering either of the interventions. These methodological limitations are reflected in the lower effect sizes obtained relative to the previously reviewed cocoa flavanol studies. Combined, these studies suggest that moderately high doses of cocoa flavanol provide benefits for cognition two hours after ingestion in young adult populations. Enhanced visual functioning accompanied some of the cognitive improvements and as the majority of cognitive tasks are visual, it is possible that this concurrent effect may also play a role in the facilitation of cognitive improvement. Further cocoa flavanol studies are required to clarify the effects of dose, the effects on different populations, and the role of vision as a potential mechanism of action.

### 3.3. Green Tea Supplementation (Epigallocatechin Gallate)

Green tea is typically administered in caffeine-free extract form. The main flavanol constituent is epigallocatechin gallate EGCG. In a double-blind, placebo controlled crossover study, Wightman *et al.* [38] administered 135 mg and 270 mg doses of EGCG extract, but observed no cognitive or mood effects for either dose. Cognitive tests included simple reaction time (SRT), serial subtraction, RVIP and Stroop, and were measured at baseline and 45 min after supplementation. Near-infrared spectroscopy (NIRS) revealed a lowering of CBF during cognitive testing following the lower dose. The authors attribute this CBF effect to previously observed vasoconstrictor properties of EGCG seen at low doses. This vasoconstrictor effect appears to contrast with vasodilatory properties previously observed for higher doses. In support of the latter, a study by Scholey *et al.* [39] observed increased alpha, beta and theta electroencephalography (EEG) activity in the frontal gyrus 2 h after a high dose of 300 mg EGCG extract. This change in brain activity was accompanied by increased feelings of calm (*d* = 0.55) and a reduction in ratings of stress (*d* = 0.64). Scholey *et al.* [39] report that similar EEG activity has been observed in studies of meditation and mindfulness suggesting a correlational link with mood. However, attention outcomes measured using the RVIP task were reported as non-significant (in a separate publication [40]). The acute effects of EGCG therefore appear restricted to mood at this stage, and then only for higher doses. Some modulation of brain activity is apparent, but further investigation is needed to understand dose-related differences in vasoreactivity, and whether or not these are likely to lead to behavioural benefits on cognitive tests.

### 3.4. Ginkgo Biloba Supplementation (Quercetin, Kaempferol and Isorhamnetin)

Ginkgo biloba is a flavonol-rich leaf extract that is typically standardised to contain 24% flavonols and 6% terpenoids (a group of bioactive plant lipids). A popular herbal supplement, it is reported anecdotally to improve blood circulation, relieve anxiety and enhance memory e.g., [41]. Despite these claims, in a double-blind crossover trial, Warot *et al.* [42] failed to observe any cognitive improvements in 12 young adults 1 h after an acute dose of 600 mg (approx. 144 mg flavonols). The task battery included choice reaction time (CRT), picture recognition, the Sternberg memory scanning task, and critical flicker fusion frequency (CFF)—a measure of vigilance. However, the small sample size may have impacted the statistical power of the study. Similarly, Subhan and Hindmarch [43] found no effect on either CFF or CRT at the same time point for doses of 120, 240 or 600 mg (approx. 29, 58 and 144 mg flavonols respectively). Significant improvements were however observed for the Sternberg task, with a decrease in reaction time observed after the 600 mg dose, and decreases in memory scanning rates observed after the 120 mg and 600 mg doses. These observations were in comparison with a placebo in a double-blind crossover design; however baseline measurements were not recorded prior to administering each intervention and only 8 participants were tested. Unfortunately there was insufficient data reported in the paper to allow calculation of effect size. Again, the small sample size may have impacted the statistical power of the study.

Nathan *et al.* [44] tested at the slightly later postprandial time of 90 min but found no significant improvements in older adults following 120 mg supplementation (approx. 29 mg flavonols) using the CDR battery and RAVLT. Yet again this was a small study (*n* = 11) with a small dose size and as such the conclusions may be statistically unreliable. A larger double-blind crossover study of young adults (*n* = 20) conducted by Kennedy *et al.* [45] observed dose- and time-related improvements, in a “speed of attention” factor derived from the CDR task battery. Effects were not apparent at 1 h postprandially but became evident at 2.5, 4 and 6 h. Cohen’s d values were *d* = 0.45, *d* = 0.45 and *d* = 0.50, respectively for a 240 mg dose (approx. 58 mg flavonols), and *d* = 1.29, *d* = 0.86 and *d* = 0.80 for a 360 mg dose (approx. 86 mg flavonols) across the time period. No effect was observed for a lower 120 mg dose (approx, 29 mg flavonols). The higher effect sizes for the 360 mg dose compared with the 240 mg dose, and the lack of any significant effect for the lowest 120 mg dose, are indicative of a dose-related response for attention. This was not the case for memory; improvements in a “quality of memory” factor were observed at 1 h and 4 h for the 120 mg dose only (*d* = 0.57 and *d* = 0.52), although trends were evident for the higher doses. A follow up study by the same authors [46] comparing ginkgo effects with panax ginseng (a plant extract rich in bioactive saponins) failed to replicate the “speed of attention” finding for an identical 360 mg ginkgo biloba dose. This inconsistency was attributed to a procedural change in the second study. They did however observe serial 7s improvements at 4 h (*d* = 0.65) and 6 h (*d* = 0.28). Immediate recall (*d* = 0.83) and delayed recall (*d* = 0.67) were also observed to improve at 6 h, as did a “Quality of memory” factor (*d* = 0.59).

Elsabagh *et al.* [47] administered a low dose of 120 mg ginkgo biloba extract (approx. 29 mg flavonols) and after 4 h observed improved attention relative to a placebo condition, using a paced auditory serial addition test (PASAT) (*d* = 0.58). Improved pattern recognition (*d* = 0.55) and a trend towards improved delayed recall were also observed. This was a well powered between-subjects study (*n* = 52) but with only a single post-ingestion time point and no baseline. The two participant groups did not differ significantly in terms of age, gender, BMI, verbal IQ, or habitual caffeine and alcohol intake, however without cognitive baseline measures, the possibility remains that group differences in cognition may simply be due to differences in the participants.

In order to consolidate the ginkgo biloba data from 3 smaller studies, Kennedy *et al.* [48] combined their data in a meta-analysis. In young adults (combined *n* = 78), a general decline in “quality of memory” observed following the placebo was attenuated at 1 h and 4 h following 120 mg ginkgo biloba supplementation. However “speed of attention” performance slowed at 1 h and 6 h relative to both baseline and placebo. The authors were unable to explain this unexpected outcome, particularly as higher doses have been shown to improve speed of attention relative to placebo. Such inconsistencies in the data highlight the need for further investigation. Overall, memory findings following ginkgo biloba supplementation appear to be relatively consistent across the larger, well powered crossover studies and mimic the timings of memory effects observed in the blueberry supplementation studies, occurring at 1 h and 4–6 h postprandially. Attention effects following ginkgo biloba appear dose-dependent and seem to occur at later time points than some other flavonoid subclasses (from 2.5 to 6 h). The effects of ginkgo biloba also appear to span several cognitive domains including attention, working memory, and episodic memory. Some of the largest cognitive effect sizes, as summarised in Table 3, are observed following ginkgo biloba. This may reflect the additional effects of terpenoids also present in the extract.

**Table 3:** A summary of statistically significant cognitive outcomes in ascending order of effect size.

Study	^Age (Years)^n	Flavonoid Dose (mg)	Cognitive Measure	Postprandial Timepoint	Effect Size (d)
* Field *et al.* [37]	^18–25^30	^cocoa^773	CRT	2 h	0.16
Lamport *et al.* [21]	^18–30^24	^citrus^71	DSST	2 h	0.30
Scholey *et al.* [35]	^18–35^30	^cocoa^520	Mental fatigue	1.5–2.5 h	^(Average)^0.33
Scholey *et al.* [35]	^18–35^30	^cocoa^994	RVIP	1.5–2.5 h	^(Average)^0.35
* Field *et al.* [37]	^18–25^30	^cocoa^773	VSWM	2 h	0.35
Watson *et al.* [30]	^18–34^36	^berry^483	RVIP	1–2.5 h	^(Average)^0.45
Kennedy *et al.* [45]	^19–24^20	^ginkgo^240	Speed of attention	2.5–6 h	^(Average)^0.47
Kennedy *et al.* [46]	^21.2 (3.9)^20	^ginkgo^360	Serial 7s	4–6 h	^(Average)^0.47
Scholey *et al.* [35]	^18–35^30	^cocoa^994	Serial 3s	1.5–2.5 h	^(Average)^0.51
Kennedy *et al.* [45]	^19–24^20	^ginkgo^120	Quality of memory	1–4 h	^(Average)^0.55
Scholey *et al.* [39]	^27.7 (9.3)^31	^tea^300	Mood	2 h	0.55
* Elsabagh *et al.* [47]	^18–26^52	^ginkgo^120	Pattern recognition	4 h	0.55
Scholey *et al.* [35]	^18–35^30	^cocoa^520	Serial 3s	1.5–2.5 h	^(Average)^0.56
Dodd [25]	^18–25^19	^berry^631	Letter memory	5 h	0.57
Dodd [25]	^62–73^18	^berry^631	Word recognition	2–5 h	^(Average)^0.57
Alharbi *et al.* [33]	^30–65^24	^citrus^272	CPT	6 h	0.58
* Elsabagh *et al.* [47]	^18–26^52	^ginkgo^120	PASAT	4 h	0.58
Kennedy *et al.* [46]	^21.2 (3.9)^20	^ginkgo^360	Quality of memory	6 h	0.59
Watson *et al.* [30]	^18–34^36	^berry^467	Digit vigilance	1–2.5 h	^(Average)^0.64
Scholey *et al.* [39]	^27.7 (9.3)^31	^tea^300	Mood	2 h	0.64
Kennedy *et al.* [46]	^21.2 (3.9)^20	^ginkgo^360	DR	6 h	0.67
* Whyte & Williams [26]	^8–10^14	^berry^143	RAVLT	2 h	0.74
Alharbi *et al.* [33]	^30–65^24	^citrus^272	Finger tapping	2–6 h	^(Average)^0.75
^#^ Caldwell *et al.* [32]	^74.1 (7.9)^5	^berry^55	Task switching	6 h	(d_b_)0.75
Whyte *et al.* [27]	^7–10^21	^berry^253	Flanker	3 h	0.78
Whyte *et al.* [27]	^7–10^21	^berry^253	Word recognition	6 h	0.78
Whyte *et al.* [27]	^7–10^21	^berry^253	IR	1.25 h	0.80
Kennedy *et al.* [46]	^21.2 (3.9)^20	^ginkgo^360	IR	6 h	0.83
Kennedy *et al.* [45]	^19–24^20	^ginkgo^360	Speed of attention	2.5–6 h	^(Average)^0.98

* Studies with no baseline measurements; ^#^ Studies with no control condition; ^(Average)^ Average of effect sizes for the same dose and cognitive measure recorded across multiple time points.

## 4. Mechanisms of Action

### 4.1. Absorption and Metabolism

There is currently little evidence to suggest that flavonoids or their metabolites are able to cross the blood brain barrier in any great quantity, however the timings of peak levels in blood plasma have been observed to correspond with the timings of cognitive effects. For example, plasma anthocyanins and their metabolites are observed to peak at 1–2 h and 6 h following blueberry supplementation [49], which seems to correspond with the two distinct timings of cognitive benefits observed for blueberry. Plasma flavanols following cocoa ingestion peak at 2 h only [50,51,52,53], and this is reflected by a number of positive cognitive effects at 2 h but an absence of cognitive effects at later time points. Conversely, the cognitive findings for ginkgo biloba are mainly observed between 4 and 6 h postprandially [45,46,47]. Bioavailability studies for ginkgo were not apparent in the literature, however bioavailability of some conjugates of quercetin, the main flavonoid present in ginkgo, have been observed to peak at 4–6 h postprandially [54]. This may also explain the lack of cognitive findings for quercetin-rich apple where cognitive testing was carried out at 2.5 h [34]. Similarly, late peak plasma timings have also been observed for the citrus flavonoid hesperidin [55]. The reviewed cognitive effects following orange juice consumption support this, but also show an earlier effect on psychomotor performance that may relate to additional mechanisms of action such as increased cerebral/peripheral blood flow. In general however, the timings of cognitive effects appear closely related to the absorption and metabolism rates of the supplemented flavonoid compounds.

### 4.2. Endothelial Function

#### 4.2.1. Vasodilation

The vasoactive properties of flavonoids as demonstrated by flow-mediated dilation of the brachial artery following ischemia (FMD) [49,50,56,57,58,59], peripheral arterial tonometry (PAT) [56], and Laser Doppler Flowmetry (LDF) [60], are known to result in increased peripheral blood flow. Peak vasodilatory effects have been observed at 1–2 h and 6 h postprandially for blueberry supplementation [49], at 2 h for cocoa [50,57], at 30 min [58] and 2 h [59] for EGCG, at 4 h for cranberry [56], and at 6 h for orange juice [60], thereby covering the full range of time points at which cognitive effects have been observed. These endothelial effects are not restricted to peripheral systems; flavonoids have also been observed to result in increased CBF 1 h after acute blueberry supplementation [25], and 2 h after cocoa [61] and orange juice supplementation [21]. Selective increases have been observed in the dorsolateral prefrontal cortex (DLPFC) following green tea supplementation [62], however time of testing and exact dose of EGCG were not stated in the paper which makes interpretation of the observation difficult. It is likely that CBF increases will occur across the range of time points observed in peripheral blood flow studies, but there are currently insufficient published findings to support this fully. In particular, little work appears to have been reported regarding CBF modulation following acute ginkgo biloba supplementation in humans. However, CBF has been shown to be positively correlated with cognitive performance, particularly in epidemiological assessment of dementia risk, where CBF is reduced in patients with dementia, and greater CBF velocity is associated with a lower rate of cognitive decline and lower risk of dementia in healthy ageing [19,63]. Cognitive training during healthy ageing has also been observed to increase both CBF and cognitive performance [64]. Therefore, vasodilatory mechanisms of action may account for at least some of the cognitive improvements observed in acute supplementation studies. Cognitive improvements following flavonoid ingestion have yet to be directly matched with acute increases in CBF, making this an important area for further research.

#### 4.2.2. Nitric Oxide Synthesis

There are a number of different chemical mediators for vasodilation including nitric oxide (NO). Flavonoids have been associated with acute augmentation of NO status. Bondonno *et al.* [34] demonstrated a significant increase in plasma NO in response to flavonoid rich apple, although no cognitive benefits were observed for a battery of tasks performed immediately after the blood samples were taken (2.5 h postprandially). Similar enhancement of NO status has been observed at 1 h for cocoa supplementation [50], and at 2 h following pure epicatechin and quercetin [65]. In addition to its role in endothelial function, NO has also been implicated in the regulation of the transcription factor CREB; an important factor in neuron survival and synaptic plasticity [66]. A proposed mechanism for flavonoid induced augmentation of NO status is through enhancing the expression or activity of endothelial nitric oxide synthase (eNOS) [67]. eNOS itself has been implicated in the regulation of BDNF expression [68]. This may account for the increased plasma BDNF levels observed after acute blueberry supplementation [25], although this connection is speculative at this stage. Depending on the isoform present, NOS has also been implicated as a causal agent in neurodegenerative disease [69,70]. Although eNOS is considered to be neuroprotective, neuronal and inducible isoforms (nNOS and iNOS) are thought to be neurotoxic through mechanisms of oxidative stress. So flavonoid regulatory effects on NO systems may be beneficial over long term supplementation, in addition to the acute mechanisms considered here.

### 4.3. Blood Glucose Regulation

Attenuation of decline in blood glucose concentrations observed following blackcurrant [30,71] and cranberry [72] suggests that blood glucose regulation may provide an additional mechanism of action for the executive function effects observed in young adults. In particular, Watson *et al.* [30] observed both higher blood glucose levels and improved attention 1 h following blackcurrant supplementation relative to a sugar matched control. There is some evidence to suggest that the absorption of sugar may be slowed when consumed in conjunction with flavonoids. Therefore a flavonoid-rich drink may result in greater availability of glucose over a longer period relative to a low flavonoid sugar matched control. As glucose is necessary for all human cell function, and glucose supplementation has been directly linked to cognitive improvement [73,74,75], this mechanism offers a plausible explanation for the observed cognitive benefits of flavonoid-rich drinks (relative to sugar matched controls) in the immediate postprandial period. Similar effects may be observed following consumption of carbohydrate rich foods such as dark chocolate, or for low carbohydrate drinks such as green tea if consumed alongside carbohydrate based foods, however further research is needed.

### 4.4. Neuronal Enhancement

#### 4.4.1. Monoamine Oxidase Inhibition

Monoamine levels, particularly for dopamine, have been observed to increase during working memory and attention tasks, correlating positively with task performance [76]. Therefore inhibition of monoamine oxidase (MAO) may be beneficial to monoaminergic neurotransmission during cognitive performance. Watson *et al.* [30] observed MAO inhibition in association with improved attention after blackcurrant supplementation. None of the other studies reviewed here have measured MAO levels, but *in vitro* studies and animal studies using chronic supplementation have demonstrated MAO inhibitory effects for all flavonoid subclasses [77]. Consideration should be given to the inclusion of such measures in future research.

#### 4.4.2. BDNF Synthesis

Trends towards increased plasma BDNF observed after 1 h [25] are a slightly unexpected finding in an acute study. BDNF regulated protein synthesis mechanisms of flavonoid action have been more consistently associated with chronic supplementation [78]. However as mentioned above, eNOS has been implicated in the regulation of BDNF expression [68]. Acute exercise has been observed to result in rapid increase in BDNF [79]. Therefore, vasodilation following flavonoid ingestion might reasonably have a similarly rapid effect on BDNF availability. Assuming BDNF levels can increase over such short time periods following flavonoid ingestion, it seems unlikely that subsequent facilitation of neuronal functioning would occur in time to explain cognitive effects apparent at only 1–2 h postprandially, but may provide a possible mechanism for the cognitive improvements observed at later time points. BDNF has been implicated in both short term memory formation and long term memory formation [80] through a permissive role in the facilitation of early long term potentiation (LTP) [81], and so increases in BDNF might be responsible for acute enhancement of episodic memory.

### 4.5. Visual Function

Flavonoids have been extensively associated with improvements in visual function both *in vitro* and *in vivo* (see Kalt *et al.* [82] for a review). Improvements in visual contrast sensitivity and detection of motion direction after acute cocoa flavanol supplementation in young adults have been described above [37]. Additionally, similar contrast sensitivity improvements have also been seen in older adults [83]. Flavonoids have also been shown to influence the focussing ability of the eyes; improvements in accommodative facility have been observed in young adults following acute cocoa, and improvements in convergence facility have been observed after acute blueberry supplementation [83]. Of the studies reviewed here only 3 have reported significant findings for non-visual tasks: RAVLT [26], PASAT [47], Simple and complex finger tapping [33]. All other significant findings have been for visually presented tasks; therefore improved vision may account for at least some of these findings. The link between enhanced vision and improved cognitive performance following flavonoid supplementation is only speculative at this stage. However, epidemiological research has identified clear associations between visual acuity and cognitive performance across multiple age groups [84]. This is likely to reflect a common underlying variable such as general health. But as a possible mechanism, enhanced visual function warrants further investigation.

## 5. Summary and Conclusions

Table 3 summarises the cognitive effects observed across all reviewed studies. Acute flavonoid-induced cognitive effects have been found across multiple cognitive domains. In particular, a number of studies report improvements for attention tasks and factors. Therefore, it is possible that improvements over a range of cognitive domains may be facilitated by general improvements in attention. The evidence suggests that the effects of flavonoids on cognitive outcomes are mediated by age. For example, executive function, working memory and psychomotor processing speed effects are apparent in young and middle aged adults. Episodic memory effects appear more prevalent in children and older adults, particularly following blueberry supplementation. This may reflect the relatively lower episodic memory performance generally observed in the very young or old when compared with adults in their cognitive prime. While the hippocampus has been shown to have the potential for improved neuronal connectivity and even neuronal growth throughout life, those at critical developmental stages may simply have greater potential for episodic memory improvement. Conversely, developmental differences in the structure and functioning of the prefrontal cortex may mean that the potential for improvement in executive function is more limited in these extreme age groups.

The studies reviewed have been primarily concerned with young adults and therefore more work is needed to determine true differences between age groups, ideally using mixed designs that allow direct comparison of different participant groups within the same study. Whatever the age group, cognitive effects are likely to be dose-dependent, as evidenced by the increasing cognitive effect sizes observed with increasing doses of ginkgo biloba [45] and the similar dose-dependent vascular effects of blueberry [49]. These potential effects of dose clearly need further investigation across all flavonoid subclasses and using a wider range of doses than those currently investigated.

The timings of cognitive effects vary depending on the flavonoid source, which may reflect differences in rates of absorption and metabolism for individual flavonoid types, or simply for different food types. The majority of observed cognitive improvements match peaks in plasma metabolite concentrations and also peaks in peripheral and cerebral blood flow. Although vasodilation is often cited as the most likely mechanism for acute flavonoid induced improvements in cognition, cognitive improvements have not yet been observed directly in conjunction with vascular-related effects. This suggests a need for studies incorporating concomitant cognitive and vascular measurements. Cognitive improvements have been observed alongside other physiological changes such as altered rates of glucose absorption [30], inhibition of MOA [30], and improved vision [37], suggesting that these factors may also be influential.

Observed physiological responses to flavonoid supplementation such as vasodilation have been consistently replicated, but cognitive findings are not as robust despite the number of moderate to large effect sizes apparent in Table 3. As discussed, methodological differences between studies are likely to partially explain the inconsistencies in cognitive observations. Comparisons between studies are often difficult due to differences in dose and flavonoid source. The design of a study may also impact on the size of any observed cognitive effects. For example, from Table 3 it can be seen that effect sizes in studies without a control [32] tend to be large, whereas those with no baseline measurements [26,37,47] tend to exhibit small or moderate effect sizes or even no effects at all [34]. From Table 3 it can also be observed that studies with very small numbers of participants tend to exhibit greater effect sizes. This may be indicative of a type 1 error (false positive outcome) as such studies have a lower statistical power to determine a true cognitive effect, and other larger studies reviewed here have generally not observed similarly large effect sizes. In crossover studies, it is also important to reduce the impact of practice-related improvements in cognitive performance that is often associated with repeated testing. It is not always clear whether this has been adequately addressed in some of the studies reviewed here [26,32]. Additionally, extensive batteries of tasks are often deployed at several different time points, but often only one or two measures prove significant. This increases the potential for type 1 error considerably. Future studies therefore also need to address these methodological issues, to improve their statistical power to reliably observe small changes in cognition.

In conclusion, the limited evidence so far suggests that this is a promising area that demands further investigation. However, in terms of design, current studies are a long way from the large scale randomised controlled trials (RCTs) that are required to build a strong and robust evidence base supporting beneficial effects of flavonoid ingestion for cognitive outcomes in the immediate postprandial period.
